# A Prospective Review on Selectable Marker-Free Genome Engineered Rice: Past, Present and Future Scientific Realm

**DOI:** 10.3389/fgene.2022.882836

**Published:** 2022-06-09

**Authors:** Rajveer Singh, Navneet Kaur, Umesh Preethi Praba, Gurwinder Kaur, Mohammad Jafar Tanin, Pankaj Kumar, Kumari Neelam, Jagdeep Singh Sandhu, Yogesh Vikal

**Affiliations:** ^1^ School of Agricultural Biotechnology, Punjab Agricultural University, Ludhiana, India; ^2^ Department of Plant Breeding and Genetics, Punjab Agricultural University, Ludhiana, India

**Keywords:** clustered regularly interspaced short palindromic repeats/crispr associated Cas9 (Crispr/Cas9), genetic engineering, genetically modified (GM) -regulation, rice, selectable marker genes (SMGs)

## Abstract

As a staple food crop, rice has gained mainstream attention in genome engineering for its genetic improvement. Genome engineering technologies such as transgenic and genome editing have enabled the significant improvement of target traits in relation to various biotic and abiotic aspects as well as nutrition, for which genetic diversity is lacking. In comparison to conventional breeding, genome engineering techniques are more precise and less time-consuming. However, one of the major issues with biotech rice commercialization is the utilization of selectable marker genes (SMGs) in the vector construct, which when incorporated into the genome are considered to pose risks to human health, the environment, and biodiversity, and thus become a matter of regulation. Various conventional strategies (co-transformation, transposon, recombinase systems, and MAT-vector) have been used in rice to avoid or remove the SMG from the developed events. However, the major limitations of these methods are; time-consuming, leftover cryptic sequences in the genome, and there is variable frequency. In contrast to these methods, CRISPR/Cas9-based marker excision, marker-free targeted gene insertion, programmed self-elimination, and RNP-based delivery enable us to generate marker-free engineered rice plants precisely and in less time. Although the CRISPR/Cas9-based SMG-free approaches are in their early stages, further research and their utilization in rice could help to break the regulatory barrier in its commercialization. In the current review, we have discussed the limitations of traditional methods followed by advanced techniques. We have also proposed a hypothesis, “DNA-free marker-less transformation” to overcome the regulatory barriers posed by SMGs.

## 1 Introduction

The green revolution has led to remarkable progress through high-yielding crop varieties worldwide. Food security is the key mandate of agriculture systems to feed the ever-exceeding global human population (expected to be 10 billion by 2050). Rice (*Oryza sativa*) is one of the major staple food crops worldwide. Asian countries constitute approximately 91% of rice, preceded by South America, North and Central America, Europe, and Oceania (Fraiture et al., 2016). However, its production has faced constant challenges due to the biotic and abiotic stresses that have emerged through climate change (Stallworth et al., 2020; Hernandez-Soto et al., 2021). Rice genetic improvement has been made through conventional breeding, molecular approaches, and genetic and genome engineering techniques to enhance yield potential and resistance to biotic and abiotic stresses (Das and Rao, 2015; Singh et al., 2020). Although molecular breeding is a leading method of crop improvement, including biotic and abiotic stresses (Waseem et al., 2021; Islam et al., 2022), during the continuous domestication and selection, significant genetic diversity has been lost (Singh et al., 2016). Moreover, breeding programs require ample time to transfer certain traits from wild relatives into elite cultivars, generally employing foreground and repeated background selections.

An alternative to these breeding strategies, genome engineering approaches represent a new way to tailor crop architecture in a comparably short time interval. At the beginning of the last decade (in the year 2013) the emergence of a new genome-editing tool, “Cluster Interspaced Short Palindromic Repeat” and its associated Cas9 nuclease (CRISPR/Cas9) has also enabled us to design the genetic architecture of rice for various traits including biotic stresses, abiotic stresses and other qualitative traits (Fiaz et al., 2021). For instance, transgenic rice expressing Dehydration-Responsive Element-Binding (DREB) genes for drought and salt tolerance (Lata and Prasad, 2011), *Cry* gene for insect resistance (Estiati, 2020) have been developed. Lectin genes such as Allium Sativum leaf lectin (ASAL) for sap-sucking insects (Yarasi et al., 2008) and *Cry1Ac::ASAL* hybrid fusion protein for multi-insect resistance (Boddupally et al., 2018) have been incorporated into different rice cultivars. Moreover, transgenes have been targeted for bacterial blight, blast, and sheath blight resistance (Sawada et al., 2004; Molla et al., 2020), nutritional traits like Golden rice enriched with beta-carotene (Paine et al., 2005), and many others, which have significantly improved its yield and quality.

Despite the great potential of genome engineering technologies, the journey of genetically engineered crops from labs to fields and finally to commercial release has been scrutinized substantially and blocked due to the socio-ethical concerns associated with their release process. Fraiture et al. (2016) have reported that the status of biotech rice is restricted to laboratory experiments or field evaluation. Garg et al. (2018) also inferred the maximum research in transgenics but minimum utilization at the commercial level. Apart from regulatory concerns of transgene expression (transgenic research) and off-target effects (genome editing research) in engineered rice, the main issue is the use of selectable marker genes (SMGs) placed next to the genetic construct in the transfer-DNA (T-DNA) region of the plasmid. *Neomycin phosphotransferase II* (*npt II*) and *hygromycin phosphotransferase* (*hpt*) are routinely used antibiotic resistance marker genes (ARMGs) (Hiei et al., 1997; Twyman et al., 2002; Breyer et al., 2014). The ARMGs present in transgenics is of no use but is of regulatory concern for the release and commercialization of transgenic crops (Breyer et al., 2014). The harness of ARMGs in transgenic plants has been questioned over the past few years as horizontal gene transfer from plant to soil bacteria or human intestinal microbes by plant products consumed as food. However, all these apprehensions are merely suppositional issues lacking scientific shreds of evidence (Ramessar et al., 2007; Breyer et al., 2014). The use of ARMGs in Genetically Modified (GM) plants is opposed strictly by many national governments, Non-Governmental Organizations (NGOs), industries, and regulators. The European Union (EU) raises concerns about the use of ARMGs and strictly opposes them in Genetically Modified Organisms (GMOs), as they may adversely affect human health and cause environmental risks (European Parliament Council of the European Union, 2001).

Alternative to selective antibiotics, second-generation non-antibiotic SMGs have also been employed in rice genetic transformation e.g., herbicide resistance gene for bialaphos (*bar*) (Rathore et al., 1993; Zhao et al., 2007). However, the use of herbicide resistant genes has several limitations related to the environment (Breyer et al., 2014). Additionally, *hpt* in Golden rice 1 (GR1) was opposed strictly due public perception of it, so new Golden rice 2 (GR2) events were developed by Syngenta. Instead of having an antibiotic marker, the *phosphomannose isomerase* (*pmi*) gene was used (Paine et al., 2005). More recently, *phosphite oxidoreductase* (*ptxd*) has been utilized as a selection marker in rice (Dormatey et al., 2021; Liu et al., 2021). A battery of scorable marker (positive selection) genes such as *gus* (*ß-glucuronidase*), *gfp* (*green fluorescent protein*), *luc* (firefly *luciferase*) and *manA* (*mannose A*) have been employed for screening transgenic rice to overcome the limitations posed by the use of antibiotics and herbicide resistant genes (Sah et al., 2014). A series of systems have been developed to avoid the use of SMGs and their removal from transgenic plants. The SMGs-free system includes co-transformation, site-specific recombinase, transposon-based, MAT (Multi Auto-Transformation) vector, DRB (Double Right Border)-binary vector, and marker-free transformation, which have been discussed in great detail in many reviews (Chong-Pérez and Angenon, 2013; Yau and Stewart, 2013; Breyer et al., 2014). The scope of the current review is not only to account in brief for these systems but also to discuss recently developed marker-free systems and their utility in developing rice free from selectable markers. Thus, it is imperative to study its current regulatory status to understand future visions for the commercialization of marker-free biotech rice.

## 2 Account on Selectable Marker Genes-Free Engineered Rice

**FIGURE 1:**
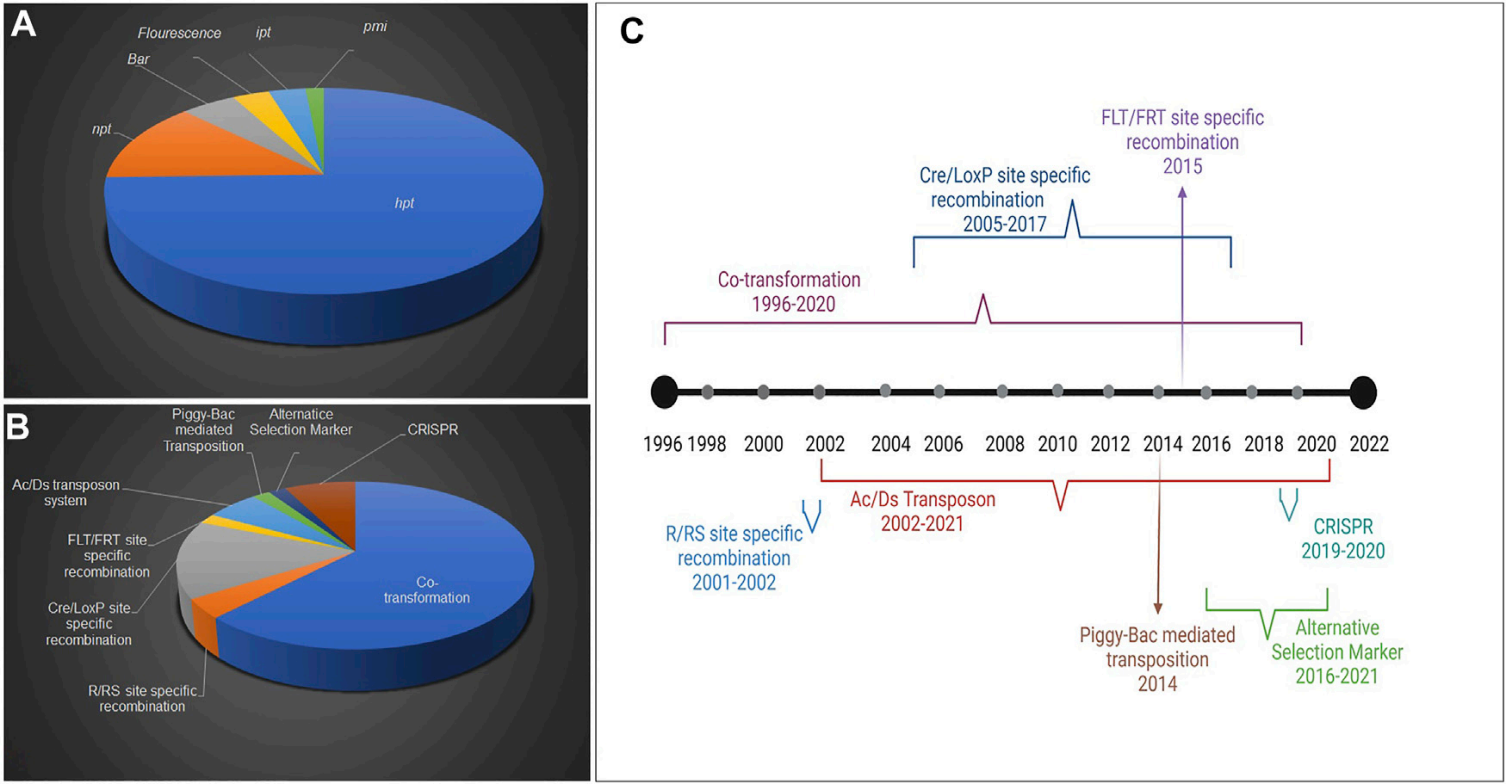
Status of selectable markers used for the generation of SMG-free transgenic rice. Representation of various selectable markers contribution **(A)**, Timeline representation of SMG-free techniques used in rice **(B)**, Proportion of different molecular approaches in developing SMG-free engineered rice **(C)**.

Plant genetic engineering would not have become possible without selectable markers. The selectable markers allow the transformed cells to grow favorably where otherwise they face competition and being overgrown by non-transformed cells. The percent use of specific selectable markers in rice is represented in Figure 1A. The study showed that the most widely used SMG is *hpt* (74.6%), followed by *npt II* (12.6%), *Bar* (4.7%), fluorescence, and *isopentyl transferase* (*ipt*) (3.1%), and *pmi* (1.5%) genes. The decline in the use of the *Bar* gene as the selectable marker is due to its positional effect and pleiotropic effect on the expression of plant genes (Miki et al., 2009). It is also worth accounting for the technique used in rice as a percentage, based on several publications (1996–2021) (Figure 1B). The co-transformation technique almost accounts for 62.2% of rice transformation, followed by site-specific recombination methods (20.5%), transposon (7.4%), and CRISPR (Clustered regularly interspaced short palindromic repeats)-based methods (7.5%). It is interesting to study the trend of various SMG-free technologies used so far from their beginning in rice. A timeline of diverse SMG-free techniques in rice has been retrieved from literature (1996–2021) and illustrated based on their year-wise use (Figure 1C). The most premier and prevalent technique used in rice is co-transformation was first reported in 1996 (Komari et al., 1996), with the most recent publication in 2018 (Rajadurai et al., 2018). It is anticipated that more publications on this subject will follow in the future. Besides co-transformation, site-specific recombination techniques including Reversible Recombinase system (R/Rs), Cyclic recombinase enzyme (Cre/lox), and Flippase/Flippase recognition target (FLP/FRT) are other methods of excising-out SMG using homologous recombination. These have been widely adopted in rice between 2001 and 2017, starting with R/Rs (2001–2002), but later on, the commonly used recombinase system was largely Cre/lox (2005–2017). However, only a single report on the FLP/FRT system use is available in rice (Woo et al., 2015). Another method of auto-excision used in rice is the transposon-based removal of SMG between 2002 and 2021. The majority of approaches used transposon system Ac/Ds (reported in five publications to date). “Piggyback” transposon from the cabbage lopper moth (discussed in the next section) was used in one study (Nishizawa-Yokoi et al., 2015).

With the dawn of CRISPR as a genome editing tool, its flexibility and versatility have allowed us to use it as a tool for removing SMG from engineered plants. Recently, there have been reports of the use of CRISPR/Cas9 to remove selectable markers using homology-directed repair (HDR) based marker excision (Dong et al., 2020), marker-free targeted gene insertion (Tan et al., 2022), and transgene-free Ribonucleoprotein (RNP) based genome editing in rice (Banakar et al., 2020). A comprehensive list of techniques used to produce SMG-free rice is shown in Table 1. The numerical data of these SMG-free techniques during their current and historical use in rice might assist with correlating their efficiency, ease, and even their regulatory aspects.

**TABLE 1:** Summary of selectable markers and techniques used to create SMG-free engineered rice.

S. no.	Method(s) used to generate SMG-free Plants	Selectable marker gene	Target gene(s)	Target Trait	References
1	Co-transformation	*HPT and NPT-II*	*GUS*	GUS activity in plant leaves	Komari et al. (1996)
2	Co-transformation	*HPT*	*uidA*	Gus activity in rice transgenic cells	Huang et al. (2001)
3	Co-transformation	*HPH, Bar*	*Rice ragged stunt virus (RRSV)*	Viral resistance	Lu et al. (2001)
4	R/RS site-specific recombination + Ac transposable elements	*HPT and NPT-II*	*R gene of zygosaccharomyces rouxii*	Generation of deletion in rice genome	Nakagawa et al.(2001)
5	Ac/Ds transposon system	*HPH*	*cry1B*	Insect resistance	Cotsaftis et al.(2002)
6	R/RS site-specific recombination	*IPT*	*Gus A, NPTII,* and *hpt*	Model genes of interest	Endo et al. (2002)
7	Co-transformation	*HPT*	*glutelin A (Antisense)*	Glutelin content in seeds	Maruta et al. (2002)
8	Co-transformation	*HPT and PMI*	*Phytoene synthase (psy), lycopene β-cyclase (lcy),* and *phytoene desaturase (crtI)*	Caroteneoid accumulation	Datta el al. (2003)
9	Co-transformation	*HPH*	*cryIAb/cryIAc*	Insect resistance (yellow stem borers and leaf-folders)	Tu et al. (2003)
10	Co-transformation	*HPT*	*bar*	Herbicide resistance	Breitler et al. (2004)
11	Co-transformation	*NPT-II and HPH*	*crtI , psy, and lyc*	Caroteneoid accumulation	Parkhi et al. (2005)
12	Cre/loxP site-specific recombination	*IPT*	*79 bp of XVE*	—	Sreekala et al. (2005)
13	Co-transformation	*HPH*	*psy, crtI, and lyc*	Accumulation of provitamin A in the endosperm tissue	Baisakh et al. (2006)
14	Cre/loxP site-specific recombination	*HPT*	*Vitreoscilla hemoglobin (VHb), trans-zeatin synthetase (tzs), and modified 5-enolpyruvylshikimate-3-phosphate synthase (EPSPS)*	—	Cao et al. (2006)
15	Co-stranformation	*HPH*	*chip (pistil chitinase )*	Pistil-predominant chitinase (blast-disease resistance)	Hashizume et al. (2006)
16	Co-transformation	*HPT*	*Amphipathic protein (APl)*	Enhanced disease resistance	Yu et al. (2006)
17	Co-transformation	*HPT*	*Xa21*	Bacterial blight (BB) resistance	Xia et al. (2006), Gao et al. (2011)
18	Co-transformation	*HPT*	*Human lactoferrin (hLF), a lysine-rich protein gene from potato (SB401), and a methionine-rich protein gene from rice (RZ10)*	—	Li et al. (2007)
19	Co-transformation	*Bar*	*CecropinB*	Resistance against a range of plant pathogenic bacteria (*Xanthomonas compestris pv oryzae*)	Zhao et al. (2007)
20	Cre/loxP site-specific recombination	*NPT-II*	*Gus controlled by OsMAD45*	Gus assay (Expression pattern of *OsMAD45* promotor)	Bai et al. (2008)
21	Co-transformation	*HPH*	*gluA-4XCII250–270*	Accumulating a type II-collagen tolerogenic peptide	Hashizume et al. (2008)
22	Co-transformation	*HPH*	*Rice chitinase (chi11)*	sheath blight resistance	Sripriya et al. (2008)
23	Co-transformation	*HPT*	*Cry1Ab*	Lepidopteran Pest Resistance	Qi et al. (2009)
24	Co-transformation	*HPT*	*cryIA(c)*	resistance to chewing insects	Yu H. X. et al. (2009)
25	Co-transformation	*HPT*	*Waxy (Wx)*	high amylose content (AC)	Yu H. et al. (2009)
26	Co-transformation	*HPT*	*cry1B-1Aa*	Insect resistance (yellow stem borer)	Kumar et al. (2010)
27	Cre/lox site-specific recombination	*HPT*	*ASAL*	Resistance to sap-sucking planthoppers	Sengupta et al. (2010)
28	Co-transformation	*HPH*	*chi11*	Sheath blight disease resistance	Ramana Rao et al. (2011)
29	Cre/loxP site-specific recombination	*NPT*	*Gus A*	GUS assay	Khattri et al. (2011)
30	Cre/loxP site-specific recombination	*NPT and HPT*	*GUS driven by maize ubiquitin promoter*	GUS activity	Nandy and Srivastava, (2012)
31	Ac/Ds transposon system	*HPT*	*partial sequences of the first intron of rice epsps*	—	Li and Charng, (2012)
32	Co-transformation	*HPT*	*inverted-repeat (IR) structures targeting the rice stripe virus (RSV) coat protein (CP) and the special-disease protein (SP)*	Resistance to rice stripe virus (RSV )	Jiang et al. (2013)
33	Co-transformation	*HPT-II*	*High Molecular Weight Glutenin Subunits (HMW-GS) Gene- Glu-1Bx*	Increasing bread-making quality	Park et al. (2013)
34	Co-transformation	*HPT*	*cry1Ab*	Insect resistance (silkworm)	Qi et al. (2013)
35	Co-transformation	*HPT*	Phytoferritin	Increase iron content	Oliva et al. (2014)
36	Piggy bac mediated transposition	*HPT*	*ALS*	Herbicide bispyribac sodium (BS)-tolerant	Nishizawa-Yokoi et al. (2015)
37	Co- transformation	*HPT-II*	*Glu-1Dy10*	Increasing quality processing of bread and noodles	Park et al. (2014)
38	Co- transformation	*HPT*	*Bt*	Insect resistance	Gao et al. (2015)
39	FLP/FRT site-specific recombination	*HPT*	*NtTC*	Enhanced seed tocopherol content	Woo et al. (2015)
40	Alternative selection marker	*HPT*	*ptxD*	Weed control in rice	Manna et al. (2016)
41	Cre/loxPsite-specific recombination	*HPT-I*	*vip3BR*	Broad-spectrum insect resistance	Pradhan et al. (2016)
42	Co-transformation	*HPT*	RNAi targeting RBSDV (rice black-streaked dwarf virus)	Developing resistance	Ahmed et al. (2017)
43	Co-transformation	*HPT*	*NmDef02 antifungal defensin.*	Resistance against phytopathogenic fungus *Sarocladium oryzae*	Perez-Bernal et al. (2017)
44	Co-transformation	*HPT*	*AmA1*	Production of essential amino acids in rice seeds	Xu et al. (2017)
45	Cre/loxPsite-specific recombination	*HPT, NPT-II, BAR*	*OsB1, OsB2, OsDFR, OsC1*	Purple endosperm	Zhu et al. (2017)
46	Co-transformation	*HPT*	*cry2AX1*	Insect resistance	Rajadurai et al. (2018)
47	CRISPR	*DsRED fluorescence*	*IAA methyltransferase (IAMT)*	The difficulty for hypocotyl reorientation under gravistimulation increased growth rate of pollen tube	Aliaga-Franco et al. (2019)
48	CRISPR-Cas9 RNP	*Hygromycin*	*DROOPING LEAF (DL)*	Drooping leaf phenotype	Toda et al. (2019)
49	Co-transformation	*HPT*	*SSSII-2*	Soft kernels	Xu et al. (2020)
50	CRISPR-Cas9 RNP (co-delivered with plasmid)	*HPT*	*PDS*	Albino phenotype	Banakar et al. (2020)
51	CRISPR-Cas9	*Hygromycin*	*SSU-crtI and ZmPsy*	Enrichment of carotenoids in seeds	Dong et al. (2020)
52	Co-transformation	*HPT*	RNAi targeting RBSDV (rice black-streaked dwarf virus)	Developing resistance	Feng et al. (2021)
53	Ac/Ds transposon system	*Green and Red Fluorescence*	*Pi21*	Rice blast disease	Li et al. (2021)
54	Alternative selection marker	*HPT,NPT II*	*ptxDq*	Catalytic activity	Liu et al. (2021)

### 2.1 Traditional Methods to Make Selectable Marker Genes-Free Rice

The foremost concern of SMGs in engineered crops is socio-ethical issues and transgene expression. Even several copies of SMGs may result in the silencing of the essential genes of plants and affect plant metabolism (Rosellini, 2012). The batteries of methods have been developed to make marker-free transgenic crops, including rice, as discussed below.

#### 2.1.1 Co-Transformation

The maximum utilization of co-transformation is due to its simplicity and safety compared to other traditional methods. This method uses two T-DNAs containing the gene of interest (GOI) and the SMG, respectively. The chance of independent integration of GOI and SMG at different loci in the plant genome allows us to eliminate SMG by simple selection in subsequent generations (Breyer et al., 2014). The integration of SMG and GOI independently could be achieved in three ways: 1) using two strains of *Agrobacterium*, each with T-DNA, one with SMG, and the other with GOI. 2) Using a single *Agrobacterium* harboring two plasmids having independent SMG and GOI. 3) Using a single plasmid carrying two independent T-DNA regions in a single *Agrobacterium*. Co-transformation has been employed successfully in many monocots and dicots (Breyer et al., 2014). The best example is GR1, where the hygromycin resistance marker gene was eliminated (Al-Babili and Beyer, 2005). Later on, marker-free *Bt* transgenic rice was generated (Woo et al., 2015).

The efficiency of co-transformation utilizing a single vector containing two T-DNAs has been linked with a high frequency of (linked co-delivery of) the target gene and marker gene and interference with non-T-DNA sequences (McCormac et al., 2001). The co-transformation method is more efficient compared to other approaches and still it is under utilization in rice to date (Xu et al., 2017; Rajadurai et al., 2018). Another modification of the co-transformation vector system is the use of a DRB binary vector system. A DRB binary vector contains two copies of T-DNA right-border (RB) sequences adjoining a selectable marker followed by a GOI and behind with a copy of the left border (LB) sequence. Two different kinds of T-DNA could be inserted, the first RB contains the SMG and the GOI together, and the second RB contains only the GOI. Consequently, these could segregate away from each other, with the progeny resulting in GOI. Lu et al. (2001) followed this method and obtained positive progeny plants with only GOI for rice ragged stunt virus (RRSV)-derived synthetic resistance gene. Similarly, Xia et al. (2006) utilized the DRB-vector technique to make marker-free and vector backbone-free transgenic rice expressing *Xa21* gene for bacterial blight disease.

#### 2.1.2 Site-Specific Recombination

Recombinase systems have also been used widely in various crops. Recombination is a well-known concept in biological systems. It occurs when two homologous sites in DNA molecules that contain a recombinase protein come together (Hirano et al., 2011). Site-specific fusion techniques in plants have been implemented to make marker-free foreign genes (Nanto and Ebinuma, 2008). The various recombinase systems (Cre-lox, FLP-FRT, and R/RS) classified under site-specific recombination are well described (Yau and Stewart, 2013). The Cre/lox system has been used to remove *hpt* and *NPT*-II in transgenic rice for the purple endosperm trait (Zhu et al., 2017). The chief limitations of recombinase systems include: 1) it is difficult to achieve 100% excision efficiency; 2) the prolonged presence of recombinase systems in the plant genome could lead to genetic and phenotypic changes making it less appealing than co-transformation; and 3) it has also been reported that chromosomal rearrangements use cryptic-target sites, and there are reports of leftover dispensable sequences of recombinase systems (Breyer et al., 2014; Nishizawa-Yokoi et al., 2015).

#### 2.1.3 MAT-Vector System

MAT vectors use oncogenes (*ipt, iaaM/H, rol*) of *Agrobacterium* as selection markers, which control the endogenous levels of plant hormones and help to regenerate transgenic cells over non-transgenic cells (Ebinuma and Komamine, 2001). In this case, the oncogenes are combined with the site-specific recombination system (*R*/RS) for transformation. Later on, the oncogenes are removed by the R/RS system to generate marker-free transgenic plants (Ebinuma et al., 2005). This system has been used to eliminate the *ipt* marker gene from the transgenic rice (Endo et al., 2002).

#### 2.1.4 Transposon-Based

Transposon-mediated transgene reintegration was used initially by Goldsbrough et al. (1993) to reposition a Dissociation (Ds) transposon-based GUS reporter gene in transgenic tomato (*Solanum lycopersicum*). The most characterized transposons belong to the *Ac/Ds* family. In this method, either GOI or SMG (present in T-DNA) is inserted between the Ds elements. Subsequently, an active transposase recognizes the Ds elements and cleaves either of them from their native position and reinserts them into another chromosomal location after the initial transformation. Later on, the SMG could be sorted out by subsequent selection (Yau and Stewart, 2013). In a few studies, this technique has been used in rice, and recently it has been used to remove selection markers in transgenic rice resistant to blast disease (Li et al., 2021). The major limitation of this technique is that it is labor-intensive to segregate out SMG from GOI, variable transposons efficiency, and they also cause mutations at an unknown site. Apart from the Ac/Ds system, another transposon named “piggyback” was used in excising the *hpt* gene from rice plants mutated for acetolactate synthase gene (ALS) using homologous recombination (HR)-mediated gene targeting (GT) (Nishizawa-Yokoi et al., 2015).

#### 2.1.5 Marker-Less Transformation

Marker-free transformation refers to transforming without SMGs. It is an ideal way to obtain marker-free GM plants. Although the frequency of recovering transgenic events is lower (2 or 3-fold) than the use of SMGs, it could vary between 1%–25% (Breyer et al., 2014). The marker-free transformation has also been achieved *via* the pollen-tube pathway, in which exogenous DNA is taken up by egg cells or zygotes after fertilization. The pollen-tube channel has been used in certain crops like cotton, wheat, maize, and rice in China (Yang et al., 2009).

### 2.2 Recent Methods Adopted to Make Selectable Marker Genes-Free Rice- CRISPR Era

Recently, the most widely used genome editing tool known as CRISPR/Cas9 has also been brought into use to remove or avoid SMG in transgenic rice. Using the CRISPR/Cas9 tool, site-specific DSB is induced at the target site, followed by a repair mechanism either through homology-directed repair (HDR) or non-homologous end joining (NHEJ). Among both, the natural occurrence of HDR is rare and thus requires a donor template to repair DSB (Zafar et al., 2020). The delivery of donor templates is quite challenging due to the difficulties of its delivery and short-time stability in the cell. Therefore, recent efforts have aimed to increase HDR efficiencies, such as geminivirus-based donor template delivery (Wang et al., 2017) and Cas9-VirD2 chimeric protein (Ali et al., 2020). HDR-based SMG excision and marker-free gene insertion have been achieved (discussed next). It is imperative to mention that CRISPR is a more precise, efficient, and less time-consuming technology. Traditional methods, like co-transformation (using two independent T-DNA plasmids), transposon and recombinase systems (which leave cryptic sequences in the host genome) need a large screening population to segregate SMG. In contrast, the CRISPR/Cas9 based SMG-free approach utilizing HDR does not leave any foreign sequences in the genome. Moreover, RNP-based genome editing is considered DNA-free, and thus does not incorporate plasmid DNA sequences in the genome. It has now become possible to get rid of selectable markers as well as transgene cassettes that persisted in the plant genomes. The utilization of CRISPR/Cas9 as an SMG-free tool has been reported in the last few years and is in infancy. However, much is expected from this technology in terms of making SMG-free rice in the future. To date, only a few studies have reported the successful use of CRISPR/Cas9 as an SMG-free technique in rice, as discussed below.

#### 2.2.1 Marker Excision

In addition to Cre/lox and Ac/Ds as auto-excision systems, CRISPR/Cas9-based HDR has been introduced as a marker excision system. Tan et al. (2022) used Pssi-driving CRISPR/Cas9-mediated HDR-based marker-free strategy (PssiCHMF) in rice. The ‘‘pssi” is a rice promoter that drives the high expression of the CRISPR/Cas9-HDR gene construct in shoot tip (containing meristem) and inflorescence to enhance homology-directed marker excision in these tissues. The Cas9 induced double-strand break (DSB) repair pathway allows the deletion of large DNA fragments. The GUS marker gene was targeted for excision using the pYLPssi::Cas9 construct with a pair of 1027-bp homology arms to improve HDR efficiency. It resulted in a 55.6% homozygous excision of marker genes, 82.2% total excision rate, and 73% of the T_0_ population showed marker excision. It is a more efficient marker excision strategy than the floral or pollen-specific promoter controlled Cre/lox systems.

#### 2.2.2 Marker-Free Targeted-Gene Insertion

Dong et al. (2020) have demonstrated the targeted insertion of carotenoid gene cassette of GR2 (lacking selectable marker gene and T-DNA border sequences) at genomic safe harbors (GSHs) site. GSHs are the regions in the genome that can accommodate transgenes without producing detrimental effects on the host organism due to genome disruption. The GSHs were the five intergenic mutation sites identified by mutant screening, which do not exhibit visible morphological changes compared with parental phenotype. The CRISPR/Cas9-based DSB followed by donor templates assisted HDR at the target location was used to insert the gene cassette. T_0_ plants were confirmed through polymerase chain reaction (PCR) for the presence of gene cassette and event (48-A7) with a golden color phenotype, which was characterized for the carotenoid using high-performance liquid chromatography (HPLC).

#### 2.2.3 Ribonucleoprotein Based Transformation

Alternative to vector-mediated genome editing, a new method of DNA-free genome editing through RNP complex introduced by Svitashev et al. (2016) in maize by targeting four genes *viz*., (*liguleless1* (*LIG*), *acetolactate synthase* (*ALS2*), and two male fertility genes (*MS26* and *MS45*). Later on, this method was adopted in many plant species such as rice, wheat, pepper, brassica, tobacco, cabbage, apple, banana, etc. (Zhang et al., 2021). The delivery method of the RNP complex in protoplast and zygote utilized polyethylene glycol (PEG) followed by electroporation. However, particle bombardment has been used in rice, wheat, and maize embryos as well as calli (Zhang et al., 2021). In the case of rice, the premier work of RNP-based genome editing has been conducted by targeting the *phytoene desaturase* (PDS) gene to test the efficiency of different Cas9 variants using particle bombardment in scutellar derived embryos (Banakar et al., 2019; Banakar et al., 2020). In RNP-based genome editing, the RNP complex could be delivered into embryos or calli either alone (SMG-free) or co-delivered with a plasmid containing a selectable marker using standard particle delivery protocol. The detailed protocol for biolistic delivery of RNP complex is discussed in maize, wheat, and rice (Svitashev et al., 2016; Liang et al., 2017; Banakar et al., 2020). The main advantage of co-delivery of RNP complex and a plasmid containing SMG is that the transformed cells grow favorably on antibiotic selection media, and transformation efficiency increases in rice embryo-derived callus (Banakar et al., 2019). Apart from embryo and callus, the primarily and widely used explant for RNP-based genome editing is the protoplast using PEG and electroporation method. The lipofectamine reagent (TransIT-2020- water-soluble cationic lipid) has been used in a few studies to deliver RNP complex in immature embryos and calli (Svitashev et al., 2016; Banakar et al., 2020).

**FIGURE 2:**
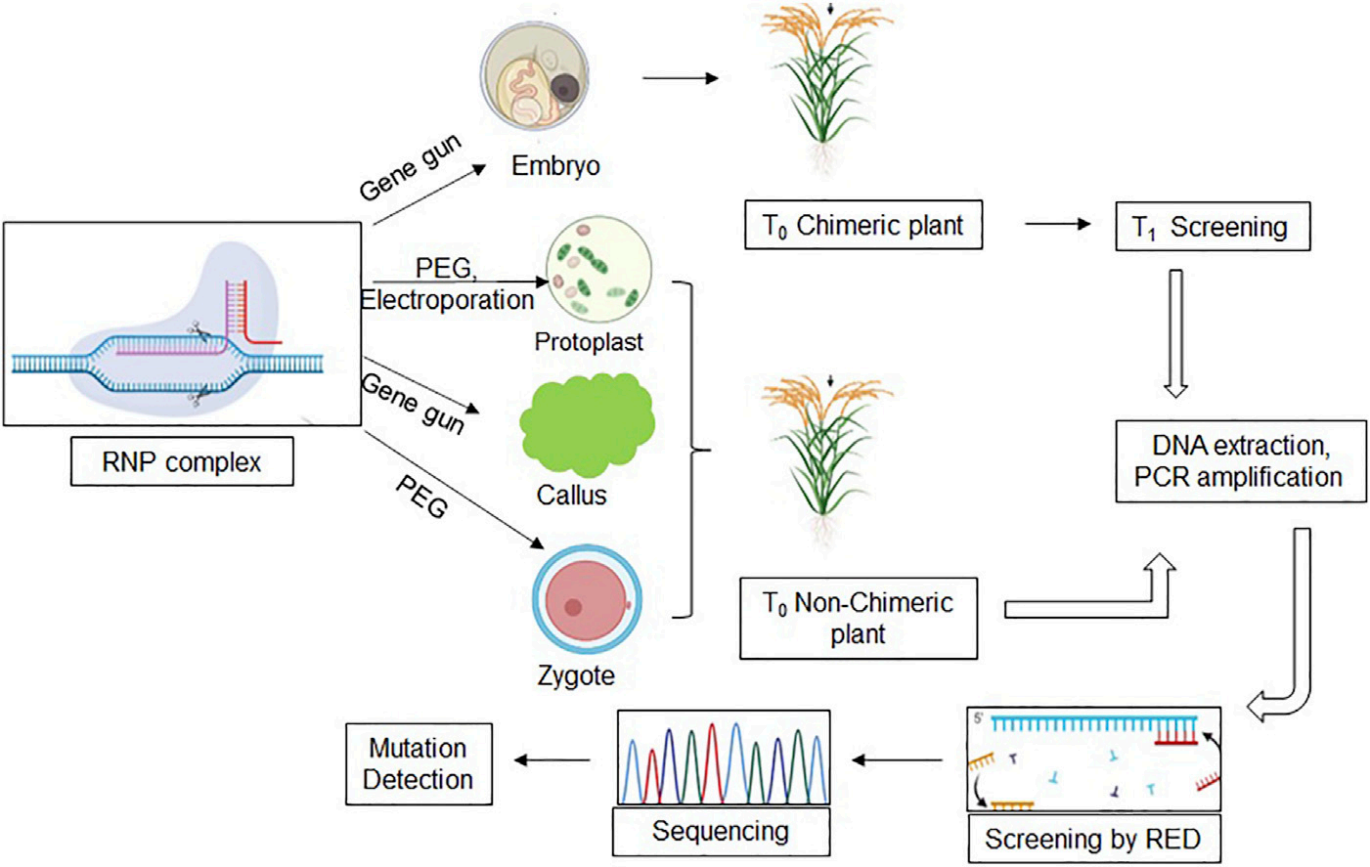
A schematic model of the CRISPR-based RNP method. The model summarizes the use of various explants (protoplast, embryo, zygote, and callus) and the protocol used for genome editing to produce SMG-free transgenic rice. RNP, (Ribonucleoprotein) complex; PEG, (Polyethylene glycol); RED, (Restriction enzyme digestion).

There are prospective reviews on the delivery methods and utilization of RNP-mediated transgene-free genome editing in various crops (Zhang et al., 2021). However, it is imperative to mention that RNP-based genome editing is challenging. It is in its starting phase, and its maximum utilization has only become possible in protoplasts, which are challenging to maintain and culture. Only a few labs have successfully utilized RNP-mediated editing versus vector-mediated genome editing (He et al., 2018). The basic workflow of RNP-based genome editing has been exhibited in various cells/tissues such as embryos, zygotes, protoplast, and callus utilizing different transformation methods (Figure 2). RNP-complex could be delivered through PEG or electroporation in protoplasts and zygotes, whereas in callus and embryo, RNP-complex could be bombarded by particle gun. It is noteworthy that T_0_ embryo transformed plants will be chimeric, and mutation must be detected in the T_1_ generation, while protoplasts, zygote, and callus-derived T_0_ plants will be non-chimeric and screened through restriction digestion and targeted sequencing.

### 2.3 Ribonucleoprotein as a Key to Success for Marker-Free Engineered Plants

The RNP complex is constituted by nuclease and guide RNA is DNA- and SMG-free approach. Earlier, RNP-based edited rice plants have been generated for grain size and grain weight (Toda et al., 2019; Banakar et al., 2020). The fragrance is considered one of the essential grain quality traits in rice as it determines the market price. The aroma in rice is associated with an increased amount of 2-acetyl-1-pyrroline (2AP) controlled by the *betaine aldehyde dehydrogenase2* (*badh2*) gene (Buttery et al., 1983). The sequence alignment of the *OsBADH2* gene among non-fragrant and fragrant lines revealed few mutations i.e., 8-bp deletion and three SNPs in exon 7, 7-bp deletion in exon 2, and 803-bp (intronic) deletions between exon 4 and 5 (Shan et al., 2015). These mutations introduce a premature stop codon upstream of key coding regions, making this gene non-functional (*badh2*) (Hashemi et al., 2013; Shan et al., 2015). A few attempts have been made to introduce aroma in non-aromatic rice through RNAi (Niu et al., 2008) and genome-editing approaches. Recently, Ashokkumar et al. (2020) successfully created novel alleles in rice variety ASD16 by knocking out the *OsBADH2* gene through a vector-based CRISPR approach.

**FIGURE 3:**
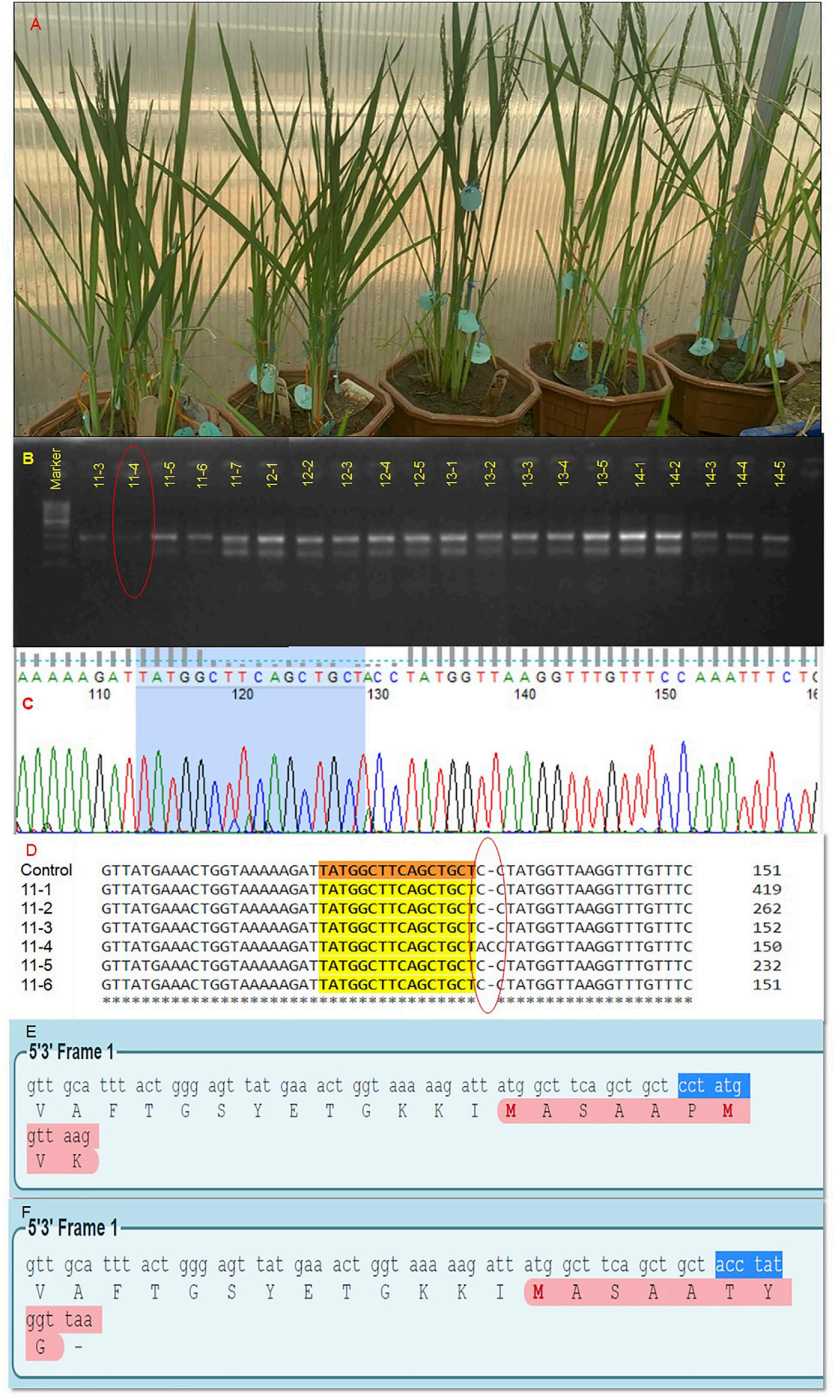
Editing of *OsBADH2* gene for generation of aromatic rice using RNP approach. Acclimatized T_0_ edited plants for *OsBADH2* gene grown under glasshouse conditions **(A)**, Detection of RNP-based editing in the T_0_ generation through mutation site based specific primers technique (MSBSP). Encircled lane depicts the mutation **(B)**, A electropherogram showing the result of Sanger sequencing **(C)**, Multiple sequence alignment of putative T_0_ plants showing the addition of a nucleotide “A” 4-bp upstream of the PAM site **(D)**, The ORF of *OsBADH2* exon seven in PR114 **(E)**, The ORF of *Osbadh2* exon seven in the edited plant, 11-4 showing change in the last four amino acid sequences indicating the disruption of protein chain **(F)**.

In our laboratory, we attempted the editing of the *OsBADH2* gene in non-aromatic rice. Basmati rice belongs to aromatic rice that has a pleasant and exquisite aroma with a low yield. However, elite cultivar PR114 lacks aroma in contrast to basmati rice. Its average yield is 6.9 tons per hectare, whereas, Basmati varieties have an average yield of 4.0 tons per hectare. The introduction of aroma in PR114 without disturbing its original genetic constitution will lead to premium quality aromatic high-yielding rice. It would lead to a major revolution for the stakeholders. A total of 1,100 embryos were bombarded by the RNP complex coated gold particles for exon 2 and exon 7 using the protocols outlined by Banakar et al. (2020). In total, 731 embryos were germinated under *in vitro* conditions on MS synthetic media, and 253 plantlets were transferred to soil. Only 35 plants survived in a glasshouse (Figure 3A), screened using the MSBSP-PCR (Mutation Site-Based Specific Primers-PCR) technique (Guo et al*.*, 2018). Seven putative edited plants were obtained through the MSBSP-PCR (Figure 3B) and were subjected to Sanger sequencing (Figure 3C). The sequences of putative edited plants were aligned against the PR114 reference sequence using Clustal Omega software, which revealed the addition of a nucleotide “A” at 4-bp upstream of PAM sequence in the target site of the edited plant # 11–4 ( Plant no. 11, tiller no. 4; Figure 3D). The alignment of the amino acid sequence of PR114 (Figure 3E) and plant 11–4 using the Expasy online tool showed the frameshift mutation in exon 7 (Figure 3F). The confirmed T_0_ plant progeny will be raised and screened through molecular and biochemical analysis. To the best of out knowledge, this is the first report on RNP-based *OsBADH2* gene editing.

### 2.4 Regulatory Perspectives

The presence of SMGs, especially *hpt*, *npt II*, and *Bar* genes in transgenic rice is one of the major hurdles in their regulatory approval. The reasons behind their strict regulations are; the spread of their resistance in natural flora and fauna, and unintended changes in plant transcriptome and metabolome (pleiotropic effect) (Chong-Pérez and Angenon, 2013). Newly developed food that is genetically modified (GM) for a particular trait has to go through rigorous testing at molecular, biochemical, and metabolic levels for food and feed safety (including toxicity, allergenicity, and anti-nutrient). This process also makes sure the claims of substantial equivalence to non-GM wild type phenotypes are valid and that the genetically modified food is safe for environmental release (Giraldo et al., 2019). Regulatory concerns related to the presence of SMGs and the importance of their withdrawal from gene cassettes needed for further approval are apparent in a few examples of GM rice events produced in the past. The first best example of transgenic rice is “Xianyou 63”, approved for release by China through co-transformation of two separate plasmids harboring *cry1Ab/Ac* and *hpt* selectable marker, respectively. The events developed were passed through the regulatory regime, and molecular characterization revealed the insertion of truncated *hpt* gene fragments (Lu, 2010). Another case is Golden rice 1 (GR1), harboring gene cassette for beta-carotene and *hpt* as a selectable marker. Event GR1 was unacceptable due to public concerns about the *hpt* marker gene. Thus, another event GR2, with a higher accumulation of beta-carotene than GR1, was produced by Syngenta using the *pmi* gene (Paine et al., 2005).

From its early development, the Golden rice trait (from GR2E event with single gene copy) has been successfully introgressed into elite rice cultivars *viz*. R64, PSBRc82, and BR29 using backcross breeding (MallikarjunaSwamy et al., 2021). After facing all the regulatory parameters, the GR2E event has been approved for consumption in different continental parts, including Australia, Canada, New Zealand, the Philippines, and the United States (https://www.goldenrice.org/). The regulation of newly developed GMOs comes under three categories. 1) process-based (for example, Europe) where the overall process or technique used to make GMO is regulated, 2) product-based (for example, the United States) where the only final product is regulated, and 3) both at the process as well as product-level regulation (for example, India). The major opponents of Golden rice are the European Union (EU), where regulation is applied to food and feed products and is a process-based regulatory scheme (https://www.ncbi.nlm.nih.gov/books/NBK424533/). Even genome-edited crops using CRISPR/Cas9 were also included in the definition of GMO as per the European Court of Justice (ECJ) in 2018 (Turnbull et al., 2021). In contrast, North America and especially the United States do not have any specific federal laws for the process regulation through which GMOs are produced. The newly developed GM products are directed to specialized regulatory bodies to assess the health, safety, and environmental laws, which are the same as those used for conventional products. In Africa, the two main approaches for seed development include biotechnology and conventional, which contribute to food and nutritional security. The former is regulated under the Biosafety act and later through the Seed act and is often accompanied by National Performance Trials (NPTs) (Akinbo et al., 2021) to ensure harmony in decision making.

### 2.5 Future Prospects

Research that aims to create SMGs-free transgenic crops has always encouraged plant molecular biologists to adopt new ways to remove selectable markers from the GM plant background. The most widely used method is co-transformation. However, it is laborious to screen a segregating population for SMG-free plants and even could not be possible in vegetatively propagated crops (Breyer et al., 2014). Alternative to the traditional methods, CRISPR/Cas9-based genetic manipulations enable the development of SMG-free crops easily and precisely. The CRISPR/Cas9 method to make SMG-free rice is at the initial stage and few attempts have been made to improve the technique. The CRISPR-based *npt* II marker degradation in transgenic tobacco has been reported by Rezaei et al. (2021) and programmed self-elimination in rice by He et al. (2018). These techniques could pave the way to making SMG-free engineered plants in the future.

There are a few limitations of the RNP-mediated genome-editing through CRISPR: 1) the low transformation efficiency of RNP; 2) the difficulty of screening plants; and 3) using embryos as an explant shows chimerism in the T_0_ stage. A novel strategy has been proposed to overcome these limitations. Studies in rice have reported that over ten distinct plasmids could be delivered together into the plant genome by particle bombardment (Chen et al., 1998). The transformation of two plasmids using a biolistic gene gun exhibited a higher frequency (85%) in contrast to a single plasmid (Hilliou et al., 1999). The co-delivery of RNP and plasmid with selectable markers is a highly beneficial technique (Banakar et al., 2019). The RNP complex can edit the target gene without the integration of CRISPR elements into the genome and reduces the number of off-targets due to transient presence. The selectable marker in the plasmid facilitates the easy selection of transformed plants. This technique combines the benefits of the targeted mutation of RNP-mediated transformation and the easy selection process of a selectable marker in a plasmid. He et al. (2018) demonstrated the technique, TKC (Trangene Killer CRISPR), for the elimination of plasmids from the mutated plant using the suicidal gene (BARNASE) under the control of REG2 promoter (expressed during the early embryo development stage).

**FIGURE 4:**
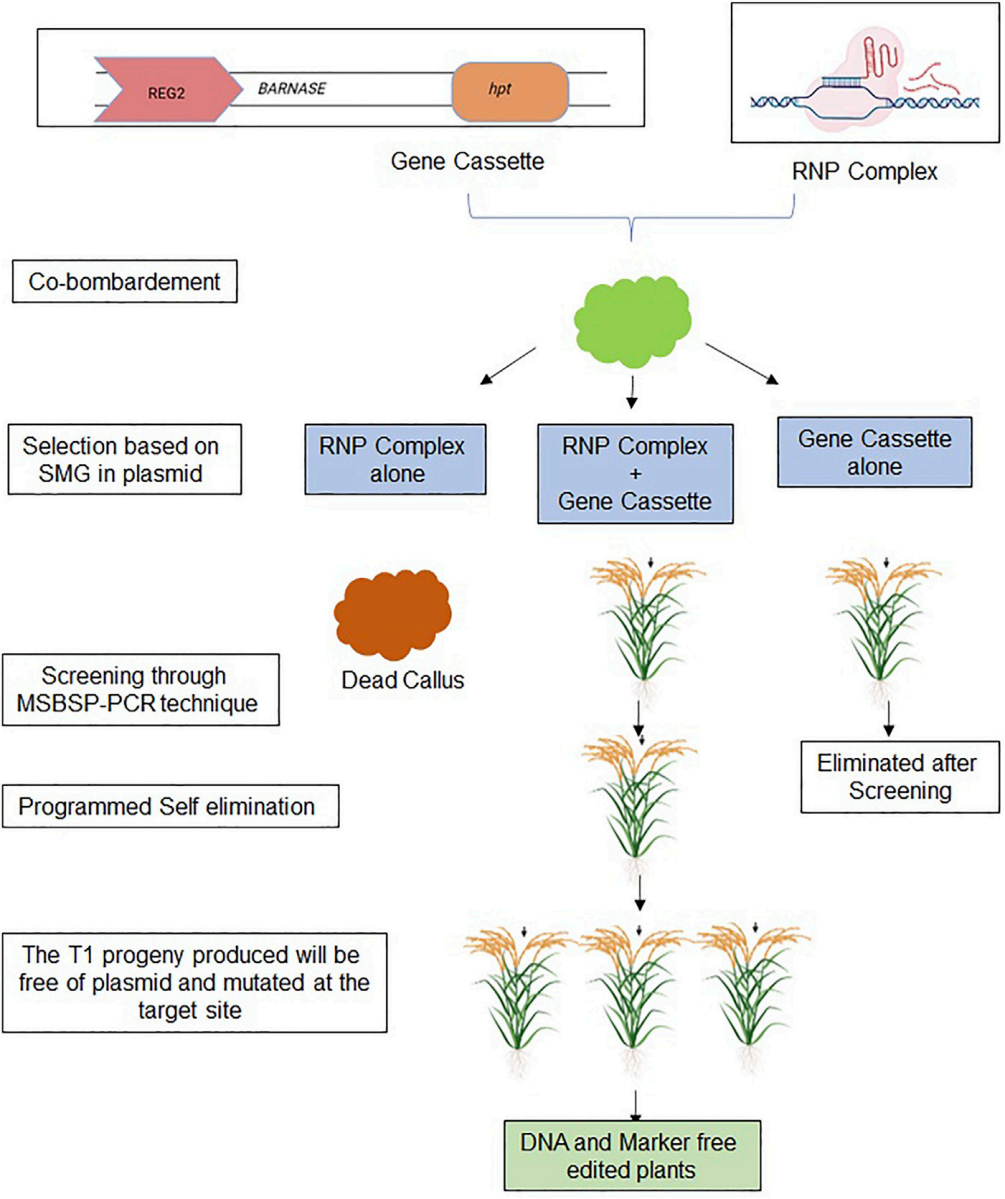
A hypothetical model for the development of DNA and marker-free genome-edited plants.

A combination of two approaches, the co-delivery of the RNP complex along with a gene cassette consisting of a suicidal gene and antibiotic-selectable markers (*hpt, npt II, etc.*) has been proposed as a new method of genome editing that is DNA and marker-free (Figure 4). In this approach, three scenarios are formed: 1) RNP transformed cells; 2) cells transformed with both RNP and cassette; and 3) cells transformed with only cassette. Transformed cells with RNP-cassette and cassette only would survive during the first screening step using selective media, while transformed plants with RNP only would be lost. In the second round of selection by MSBSP-PCR, the T_0_ plants with cassette only would be eliminated. The plants with both RNP and cassette would be selected and advance to the next generation. When these screened plants reach the seed setting stage, embryos with cassette would be killed as per the Programmed Self elimination effect, whereas plants with mutated target site lacking cassette would survive. Hence, seeds obtained from the T_0_ generation would be DNA and marker-free edited plants. We hypothesize that a straightforward and novel approach to making marker-free engineered crops for food security will support developing countries in introducing the product, thus contributing to the prologue of these products all over the world.

## References

[B1] Ahmed M. M. S. Bian S. Wang M. Zhao J. Zhang B. Liu Q. (2017). RNAi-Mediated Resistance to Rice Black-Streaked Dwarf Virus in Transgenic Rice. Transgenic Res. 26, 197–207. 10.1007/s11248-016-9999-4 27900537

[B2] Akinbo O. Obukosia S. Ouedraogo J. Sinebo W. Savadogo M. Timpo S. (2021). Commercial Release of Genetically Modified Crops in Africa: Interface between Biosafety Regulatory Systems and Varietal Release Systems. Front. Plant Sci. 12, 314. 10.3389/fpls.2021.605937 PMC8020716 33828569

[B3] Albabili S. Beyer P. (2005). Golden Rice - Five Years on the Road - Five Years to Go? Trends Plant Sci. 10 (12), 565–573. 10.1016/j.tplants.2005.10.006 16297656

[B4] Ali Z. Shami A. Sedeek K. Kamel R. Alhabsi A. Tehseen M. (2020). Fusion of the Cas9 Endonuclease and the VirD2 Relaxase Facilitates Homology-Directed Repair for Precise Genome Engineering in Rice. Commun. Biol. 3, 1–13. 10.1038/s42003-020-0768-9 31974493 PMC6978410

[B5] Aliaga-Franco N. Zhang C. Presa S. Srivastava A. K. Granell A. Alabadí D. (2019). Identification of Transgene-free CRISPR-Edited Plants of Rice, Tomato, and Arabidopsis by Monitoring DsRED Fluorescence in Dry Seeds. Front. Plant Sci. 10 (10), 1150. 10.3389/fpls.2019.01150 31620160 PMC6759815

[B6] Ashokkumar S. Jaganathan D. Ramanathan V. Rahman H. Palaniswamy R. Kambale R. (2020). Creation of Novel Alleles of Fragrance Gene OsBADH2 in Rice through CRISPR/Cas9 Mediated Gene Editing. PloS One 15 (8), e0237018. 10.1371/journal.pone.0237018 32785241 PMC7423090

[B7] Bai X. Wang Q. Chu C. (2008). Excision of a Selective Marker in Transgenic Rice Using a Novel Cre/loxP System Controlled by a Floral Specific Promoter. Transgenic Res. 17 (6), 1035–1043. 10.1007/s11248-008-9182-7 18437520

[B8] Baisakh N. Rehana S. Rai M. Oliva N. Tan J. Mackill D. J. (2006). Marker-free Transgenic (MFT) Near-Isogenic Introgression Lines (NIILs) of 'golden' Indica Rice (Cv. IR64) with Accumulation of Provitamin A in the Endosperm Tissue. Plant Biotechnol. J. 4 (4), 467–475. 10.1111/j.1467-7652.2006.00196.x 17177811

[B9] Banakar R. Eggenberger A. L. Lee K. Wright D. A. Murugan K. Zarecor S. (2019). High-frequency Random DNA Insertions upon Co-delivery of CRISPR-Cas9 Ribonucleoprotein and Selectable Marker Plasmid in Rice. Sci. Rep. 9 (1), 19902. 10.1038/s41598-019-55681-y 31882637 PMC6934568

[B10] Banakar R. Schubert M. Collingwood M. Vakulskas C. Eggenberger A. L. Wang K. (2020). Comparison of CRISPR-Cas9/Cas12a Ribonucleoprotein Complexes for Genome Editing Efficiency in the Rice Phytoene Desaturase (OsPDS) Gene. Rice 13 (1), 4. 10.1186/s12284-019-0365-z 31965382 PMC6973557

[B11] Boddupally D. Tamirisa S. Gundra S. R. Vudem D. R. Khareedu V. R. (2018). Expression of Hybrid Fusion Protein (Cry1Ac::ASAL) in Transgenic Rice Plants Imparts Resistance against Multiple Insect Pests. Sci. Rep. 8 (1), 8458. 10.1038/s41598-018-26881-9 29855556 PMC5981619

[B12] Breitler J.-C. Meynard D. Boxtel J. V. Royer M. Bonnot F. Cambillau L. (2004). A Novel Two T-DNA Binary Vector Allows Efficient Generation of Marker-free Transgenic Plants in Three Elite Cultivars of Rice (Oryza Sativa L.). Transgenic Res. 13 (3), 271–287. 10.1023/b:trag.0000034626.22918.0a 15359604

[B13] Breyer D. Kopertekh L. Reheul D. (2014). Alternatives to Antibiotic Resistance Marker Genes forIn VitroSelection of Genetically Modified Plants - Scientific Developments, Current Use, Operational Access and Biosafety Considerations. Crit. Rev. Plant Sci. 33 (4), 286–330. 10.1080/07352689.2013.870422

[B14] Buttery R. G. Ling L. C. Juliano B. O. Turnbaugh J. G. (1983). Cooked Rice Aroma and 2-Acetyl-1-Pyrroline. J. Agric. Food Chem. 31 (4), 823–826. 10.1021/jf00118a036

[B15] Cao M.-X. Huang J.-Q. Yao Q.-H. Liu S.-J. Wang C.-L. Wei Z.-M. (2006). Site-specific DNA Excision in Transgenic Rice with a Cell-Permeable Cre Recombinase. Mb 32 (1), 055–064. 10.1385/MB:32:1:055 16382182

[B16] Chen L. Marmey P. Taylor N. J. Brizard J.-P. Espinoza C. D'Cruz P. (1998). Expression and Inheritance of Multiple Transgenes in Rice Plants. Nat. Biotechnol. 16 (11), 1060–1064. 10.1038/3511 9831036

[B17] Chong-Prez B. Angeno G. (2013). “Strategies for Generating Marker-free Transgenic Plants,” in Genetic Engineering. Editor Idah S. N.. Intechopen, 17–48. 10.5772/55573

[B18] Cotsaftis O. Sallaud C. Breitler J. C. Meynard D. Greco R. Pereira A. (2002). Transposon-mediated Generation of T-DNA-And Marker-free Rice Plants Expressing a Bt Endotoxin Gene. Mol. Breed. 10 (3), 165–180. 10.1023/A:1020380305904

[B19] Das G. Rao G. J. N. (2015). Molecular Marker Assisted Gene Stacking for Biotic and Abiotic Stress Resistance Genes in an Elite Rice Cultivar. Front. Plant Sci. 6, 698. 10.3389/fpls.2015.00698 26483798 PMC4588116

[B20] Datta K. Baisakh N. Oliva N. Torrizo L. Abrigo E. Tan J. (2003). Bioengineered 'golden' Indica Rice Cultivars with β-carotene Metabolism in the Endosperm with Hygromycin and Mannose Selection Systems. Plant Biotechnol. J. 1 (2), 81–90. 10.1046/j.1467-7652.2003.00015.x 17147745

[B21] Dong O. X. Yu S. Jain R. Zhang N. Duong P. Q. Butler C. (2020). Marker-free Carotenoid-Enriched Rice Generated through Targeted Gene Insertion Using CRISPR-Cas9. Nat. Commun. 11 (1), 1–10. 10.1038/s41467-020-14981-y 32132530 PMC7055238

[B22] Dormatey R. Sun C. Ali K. Fiaz S. Xu D. Calderón-Urrea A. 2021). ptxD/Phi as Alternative Selectable Marker System for Genetic Transformation for Bio-Safety Concerns: a Review. PeerJ. 9, e11809. 10.7717/peerj.11809 34395075 PMC8323600

[B23] Ebinuma H. Komamine A. (2001). Mat (Multi-Auto-Transformation) Vector System. The Oncogenes of Agrobacterium as Positive Markers for Regeneration and Selection of Marker-free Transgenic plantsThe Oncogenes of Agrobacterium as Positive Markers for Regeneration and Selection of Marker-free Transgenic Plants. *In Vitro* Cell. Vitro Cell.Dev.Biol.-Plant. 37, 103–113. 10.1007/s11627-001-0021-2

[B24] Ebinuma H. Sugita K. Endo S. Matsunaga E. Yamada K. (2005). Elimination of Marker Genes from Transgenic Plants Using MAT Vector Systems. Methods Mol. Biol. 286, 237–254. 10.1385/1-59259-827-7:237 15310926

[B25] Endo S. Sugita K. Sakai M. Tanaka H. Ebinuma H. (2002). Single-step Transformation for Generating Marker-free Transgenic Rice Using the Ipt-type MAT Vector System. Plant J. 30 (1), 115–122. 10.1046/j.1365-313x.2002.01272.x 11967098

[B26] Estiati A. (2020). Development of Bt Rice Potential for Yellow Stem Borer Control. J. Crop Sci. Biotechnol. 23, 395–403. 10.1007/s12892-020-00025-w

[B27] European Parliament Council of the European Union (2001). Directive 2001/18/EC of the European Parliament and of the Council of 12 March 2001 on the Deliberate Release into the Environment of Genetically Modified Organism and Repealing Council Directive 90/220/EEC. Brussels: off. J. Eur. Comm. L106, 1–38.

[B28] Feng Z. Yuan M. Zou J. Wu L. B. Wei L. Chen T. (2021). Development of Marker‐free Rice with Stable and High Resistance to Rice Black‐streaked Dwarf Virus Disease through RNA Interference. Plant Biotechnol. J. 19 (2), 212–214. 10.1111/pbi.13459 32741105 PMC7868976

[B29] Fiaz S. Ahmar S. Saeed S. Riaz A. Mora-Poblete F. Jung K.-H. (2021). Evolution and Application of Genome Editing Techniques for Achieving Food and Nutritional Security. Ijms 22 (11), 5585. 10.3390/2Fijms2211558510.3390/ijms22115585 34070430 PMC8197453

[B30] Fraiture M.-A. Roosens N. H. C. Taverniers I. De Loose M. Deforce D. Herman P. (2016). Biotech Rice: Current Developments and Future Detection Challenges in Food and Feed Chain. Trends Food Sci. Technol. 52, 66–79. 10.1016/j.tifs.2016.03.011

[B31] Gao L. Xia Z. Jiang G. Peng H. Zhao X. Zhai W. (2011). Generation of Marker-free, Bacterial Blight-Resistant Transgenic Sterile Line and Hybrid Rice with Xa21. Plant Breed. 130 (4), 438–443. 10.1111/j.1439-0523.2011.01847.x

[B32] Gao X. Zhou J. Li J. Zou X. Zhao J. Li Q. (2015). Efficient Generation of Marker-free Transgenic Rice Plants Using an Improved Transposon-Mediated Transgene Reintegration Strategy. Plant Physiol. 167 (1), 11–24. 10.1104/pp.114.246173 25371551 PMC4280998

[B33] Garg M. Sharma N. Sharma S. Kapoor P. Kumar A. Ch V. (2018). Biofortified Crops Generated by Breeding, Agronomy, and Transgenic Approaches Are Improving Lives of Millions of People Around the World. Front. Nutr. 5, 12. 10.3389/fnut.2018.00012 29492405 PMC5817065

[B34] Giraldo P. A. Shinozuka H. Spangenberg G. C. Cogan N. O. Smith K. F. (2019). Safety Assessment of Genetically Modified Feed: Is There Any Difference from Food? Front. Plant Sci. 10, 1592. 10.3389/fpls.2019.01592 31921242 PMC6918800

[B35] Goldsbrough A. Lastrella C. Yoder J. (1993). Transposition Mediated Repositioning and Subsequent Elimination of Marker Genes from Transgenic Tomato. Nat. Biotechnol. 11, 1286–1292. 10.1038/nbt1193-1286

[B36] Guo J. Li K. Jin L. Xu R. Miao K. Yang F. (2018). A Simple and Cost-Effective Method for Screening of CRISPR/Cas9-induced Homozygous/biallelic Mutants. Plant Methods 14 (1), 1–10. 10.1186/s13007-018-0305-8 29872452 PMC5972395

[B37] Hashemi F. G. Rafii M. Y. Ismail M. R. Mahmud T. M. M. Rahim H. A. Asfaliza R. (2013). Biochemical, Genetic and Molecular Advances of Fragrance Characteristics in Rice. Crit. Rev. Plant Sci. 32 (6), 445–457. 10.1080/07352689.2013.807716

[B38] Hashizume F. Hino S. Kakehashi M. Okajima T. Nadano D. Aoki N. (2008). Development and Evaluation of Transgenic Rice Seeds Accumulating a Type II-Collagen Tolerogenic Peptide. Trans. Res. 17 (6), 1117–1129. 10.1007/s11248-008-9187-2 18563612

[B39] Hashizume F. Nakazaki T. Tsuchiya T. Matsuda T. (2006). Effectiveness of Genotype-Based Selection in the Production of Marker-free and Genetically Fixed Transgenic Lineages: Ectopic Expression of a Pistil Chitinase Gene Increases Leaf-Chitinase Activity in Transgenic Rice Plants without Hygromycin-Resistance Gene. Plant Biotechnol. 23 (4), 349–356. 10.5511/plantbiotechnology.23.349

[B40] He Y. Zhu M. Wang L. Wu J. Wang Q. Wang R. (2018). Programmed Self-Elimination of the CRISPR/Cas9 Construct Greatly Accelerates the Isolation of Edited and Transgene-free Rice Plants. Mol. plant 11 (9), 1210–1213. 10.1016/j.molp.2018.05.005 29857174

[B41] Hernandez-Soto A. Echeverria-Beirute F. Abdelnour-Esquivel Ana. Valdez M. Boch J. Gatica-Arias A. (2021). Rice Breeding in the New Era: Comparison of Useful Agronomic Traits. Curr. Plant Biol. 27 (7052), 100211. 10.1016/j.cpb.2021.100211

[B42] Hiei Y. Komari T. Kubo T. (1997). Transformation of Rice Mediated by Agrobacterium Tumefaciens. Plant Mol. Biol. 35, 205–218. 10.1023/A:100584761549310.1007/978-94-011-5794-0_20 9291974

[B43] Hilliou F. Christou P. Leech M. J. (1999). Development of an Efficient Transformation System for Catharanthus Roseus Cell Cultures Using Particle Bombardment. Plant Sci. 140 (2), 179–188. 10.1016/S0168-9452(98)00225-8

[B44] Hirano N. Muroi T. Takahashi H. Haruki M. (2011). Site-specific Recombinases as Tools for Heterologous Gene Integration. Appl. Microbiol. Biotechnol. 92, 227–239. 10.1007/s00253-011-3519-5 21822899

[B45] Huang N. Wu L. Nandi S. Bowman E. Huang J. Sutliff T. (2001). The Tissue-specific Activity of a Rice Beta-Glucanase Promoter (Gns9) Is Used to Select Rice Transformants. Plant Sci. 161 (3), 589–595. 10.1016/S0168-9452(01)00447-2

[B46] Islam M. R. Naveed S. A. Zhang Y. Li Z. Zhao X. Fiaz S. (2022). Identification of Candidate Genes for Salinity and Anaerobic Tolerance at the Germination Stage in Rice by Genome-wide Association Analyses. Front. Genet. 8. 10.3389/fgene.2022.822516 PMC8905349 35281797

[B47] Jiang Y. Sun L. Jiang M. Li K. Song Y. Zhu C. (2013). Production of Marker-free and RSV-Resistant Transgenic Rice Using a Twin T-DNA System and RNAi. J. Biosci. 38 (3), 573–581. 10.1007/s12038-013-9349-0 23938389

[B48] Khattri A. Nandy S. Srivastava V. (2011). Heat-inducible Cre-Lox System for Marker Excision in Transgenic Rice. J. Biosci. 36 (1), 37–42. 10.1007/s12038-011-9010-8 21451246

[B49] Komari T. Hiei Y. Saito Y. Murai N. Kumashiro T. (1996). Vectors Carrying Two Separate T‐DNAs for Co‐transformation of Higher Plants Mediated by Agrobacterium Tumefaciens and Segregation of Transformants Free from Selection Markers. Plant J. 10 (1), 165–174. 10.1046/j.1365-313x.1996.10010165.x 8758986

[B50] Kumar S. Arul L. Talwar D. (2010). Generation of Marker-free Bt Transgenic Indica Rice and Evaluation of its Yellow Stem Borer Resistance. J. Appl. Genet. 51 (3), 243–257. 10.1007/BF03208854 20720299

[B51] Lata C. Prasad M. (2011). Role of DREBs in Regulation of Abiotic Stress Responses in Plants. J. Exp. Bot. 62 (14), 4731–4748. 10.1093/jxb/err210 21737415

[B52] Li K. T. Charng Y. C. (2012). The Use of Hygromycin Phosphotransferase Gene (Hpt ) with an Artificial Intron to Obtain Marker-Off Transgenic Plants. Afr. J. Biotechnol. 11 (6), 1330–1336. 10.5897/AJB11.2198

[B53] Li X. Pan L. Bi D. Tian X. Li L. Xu Z. (2021). Generation of Marker-free Transgenic Rice Resistant to Rice Blast Disease Using Ac/Ds Transposon-Mediated Transgene Reintegration System. Front. Plant Sci. 12, 644437. 10.3389/fpls.2021.644437 33959140 PMC8095379

[B54] Li Z. H. U. Fu Y. P. Liu W. Z. Hu G. C. Si H. M. Tang K. X. (2007). Rapid Generation of Selectable Marker-free Transgenic Rice with Three Target Genes by Co-transformation and Anther Culture. Rice Sci. 14 (4), 239–246. 10.1016/S1672-6308(08)60001-3

[B55] Liang Z. Chen K. Li T. Zhang Y. Wang Y. Zhao Q. (2017). Efficient DNA-free Genome Editing of Bread Wheat Using CRISPR/Cas9 Ribonucleoprotein Complexes. Nat. Commun. 8, 14261. 10.1038/ncomms14261 28098143 PMC5253684

[B56] Liu T. Yuan L. Deng S. Zhang X. Cai H. Ding G. (2021). Improved the Activity of Phosphite Dehydrogenase and its Application in Plant Biotechnology. Front. Bioeng. Biotechnol. 9, 764188. 10.3389/fbioe.2021.764188 34900961 PMC8655118

[B57] Lu C. (2010). The First Approved Transgenic Rice in China. Gm. Crops 1 (3), 113–115. 10.4161/gmcr.1.3.12377 21865866

[B58] Lu H. J. Zhou X. R. Gong Z. X. Upadhyaya N. M. (2001). Generation of Selectable Marker-free Transgenic Rice Using Double Right-Border (DRB) Binary Vectors. Aust. J. Plant Physiol. 28, 241–248. 10.1071/PP00129

[B59] MallikarjunaSwamy B. P. Marundan S. Samia M. Ordonio R. L. Rebong D. B. Miranda R. (2021). Development and Characterization of GR2E Golden Rice Introgression Lines. Sci. Rep. 11 (1), 1–12. 10.1038/s41598-021-82001-0 33510272 PMC7843986

[B60] Manna M. Achary V. M. M. Islam T. Agrawal P. K. Reddy M. K. (2016). The Development of a Phosphite-Mediated Fertilization and Weed Control System for Rice. Sci. Rep. 6 (1), 1–12. 10.1038/srep24941 27109389 PMC4842969

[B61] Maruta Y. Ueki J. Saito H. Nitta N. Imaseki H. (2002). Transgenic Rice with Reduced Glutelin Content by Transformation with Glutelin A Antisense Gene. Mol. Breed. 8 (4), 273–284. 10.1023/A:1015208517669

[B62] McCormac A. C. Fowler M. R. Chen D. F. Elliott M. C. (2001). Efficient Co-transformation of Nicotiana Tabacum by Two Independent T-DNAs, the Effect of T-DNA Size, and Implications for Genetic Separation. Trans. Res. 10, 143–155. 10.1023/A:1008909203852 11305361

[B63] Miki B. Abdeen A. Manabe Y. MacDonald P. (2009). Selectable Marker Genes and Unintended Changes to the Plant Transcriptome. Plant Biotechnol. J. 7 (3), 211–218. 10.1111/j.1467-7652.2009.00400.x 19261135

[B64] Molla K. A. Karmakar S. Molla J. Bajaj P. Varshney R. K. Datta S. K. (2020). Understanding Sheath Blight Resistance in Rice: the Road behind and the Road Ahead. Plant Biotechnol. J. 18 (4), 895–915. 10.1111/pbi.13312 31811745 PMC7061877

[B65] Nakagawa Y. Machida C. Machida Y. Toriyama K. (2001). A System to Induce the Deletion of Genomic Sequences Using R/RS Site-specific Recombination and the Ac Transposon in Transgenic Rice Plants. Theor. Appl. Genet. 102 (8), 1136–1141. 10.1007/s001220100580

[B66] Nandy S. Srivastava V. (2012). Marker‐free Site‐specific Gene Integration in Rice Based on the Use of Two Recombination Systems. Plant Biotech. J. 10 (8), 904–912. 10.1111/j.1467-7652.2012.00715.x 22686401

[B67] Nanto K. Ebinuma H. (2008). Marker-free Site-specific Integration Plants. Trans. Res. 17, 337–344. 10.1007/s11248-007-9106-y 17588210

[B68] Nishizawa-Yokoi A. Endo M. Ohtsuki N. Saika H. Toki S. (2015). Precision Genome Editing in Plants via Gene Targeting and piggyBac-Mediated Marker Excision. Plant J. 81 (1), 160–168. 10.1111/tpj.12693 25284193 PMC4309413

[B69] Niu X. Tang W. Huang W. Ren G. Wang Q. Luo D. (2008). RNAi- Directed Downregulation of OsBADH2 Results in Aroma (2-Acetyl-1-Pyrroline) Production in Rice (Oryza Sativa L.). BMC Plant Biol. 8, 100. 10.1186/1471-2229-8-100 18840300 PMC2588449

[B70] Oliva N. Chadha-Mohanty P. Poletti S. Abrigo E. Atienza G. Torrizo L. (2014). Large-scale Production and Evaluation of Marker-free Indica Rice IR64 Expressing Phytoferritin Genes. Mol. Breed. 33 (1), 23–37. 10.1007/s11032-013-9931-z 24482599 PMC3890568

[B71] Paine J. Shipton C. Chaggar S. Howells R. M. Kennedy M. J. Vernon J. (20052005). Improving the Nutritional Value of Golden Rice through Increased Pro-vitamin A Content. Nat. Biotechnol. 23, 482–487. 10.1038/nbt1082 15793573

[B72] Park S. K. Shin D. Hwang W. H. Hur Y. J. Kim T. H. Oh S. Y. (2014). Development of Marker-free Transgenic Rice Expressing the Wheat Storage Protein, Glu-1Dy10, for Increasing Quality Processing of Bread and Noodles. J. Life Sci. 24 (6), 618–625. 10.5352/JLS.2014.24.6.618

[B73] Park S. K. Shin D. Hwang W. H. Oh S. Y. Cho J. H. Han S. I. (2013). Development of Marker-free Transgenic Rice for Increasing Bread-Making Quality Using Wheat High Molecular Weight Glutenin Subunits (HMW-GS) Gene. J. Life Sci. 23 (11), 1317–1324. 10.5352/JLS.2013.23.11.1317

[B74] Parkhi V. Rai M. Tan J. Oliva N. Rehana S. Bandyopadhyay A. (2005). Molecular Characterization of Marker-free Transgenic Lines of Indica Rice that Accumulate Carotenoids in Seed Endosperm. Mol. Genet. Genom. 274 (4), 325–336. 10.1007/s00438-005-0030-7 16179991

[B75] Pérez-Bernal M. Delgado M. Cruz A. Abreu D. Valdivia O. Armas R. (2017). Marker-free Transgenic Rice Lines with a Defensin Gene Are Potentially Active against Phytopathogenic Fungus Sarocladium Oryzae. Acta Phytopathol. Entomol. hung. 52 (2), 135–144. 10.1556/038.52.2017.021

[B76] Pradhan S. Chakraborty A. Sikdar N. Chakraborty S. Bhattacharyya J. Mitra J. (2016). Marker-free Transgenic Rice Expressing the Vegetative Insecticidal Protein (Vip) of Bacillus Thuringiensis Shows Broad Insecticidal Properties. Planta 244 (4), 789–804. 10.1007/s00425-016-2535-1 27165311

[B77] Qi Y. B. Ye S. H. Lu Y. T. Jin Q. S. Zhang X. M. (2009). Development of Marker-free Transgenic Cry1Ab Rice with Lepidopteran Pest Resistance by Agrobacterium Mixture-Mediated Co-transformation. Rice Sci. 16 (3), 181–186. 10.1016/S1672-6308(08)60077-3

[B78] Qi Y. Chen L. He X. Jin Q. Zhang X. He Z. (2013). Marker‐free, Tissue‐specific Expression of Cry1Ab as a Safe Transgenic Strategy for Insect Resistance in Rice Plants. Pest Manang. Sci. 69 (1), 135–141. 10.1002/ps.3379 22927237

[B79] Rajadurai G. Kalaivani A. Varanavasiyappan S. Balakrishnan N. Udayasuriyan V. Sudhakar D. (2018). Generation of Insect-Resistant Marker-free Transgenic Rice with a Novel cry2AX1 Gene. Electron. J. Plant Breed. 9 (2), 723–732. 10.5958/0975-928X.2018.00086.8

[B80] Ramana Rao M. V. Parameswari C. Sripriya R. Veluthambi K. (2011). Transgene Stacking and Marker Elimination in Transgenic Rice by Sequential Agrobacterium-Mediated Co-transformation with the Same Selectable Marker Gene. Plant Cell Rep. 30 (7), 1241–1252. 10.1007/s00299-011-1033-y 21327387

[B81] Ramessar K. Peremarti A. Gomez-Galera S. Naqvi S. Moralejo M. Munoz P. (2007). Biosafety and Risk Assessment Framework for Selectable Marker Genes in Transgenic Crop Plants: a Case of the Science Not Supporting the Politics. Trans. Res. 16, 261–280. 10.1007/s11248-007-9083-1 17436060

[B82] Rathore K. S. Chowdhury V. K. Hodges T. K. (1993). Use of Bar as a Selectable Marker Gene and for the Production of Herbicide-Resistant Rice Plants from Protoplasts. Plant Mol. Biol. 21, 871–884. 10.1007/BF00027118 8467080

[B83] Rezaei A. Farsi M. Malekzadeh-Shafaroudi S. Seifi A. (2021). In Planta Removal of nptII Selectable Marker Gene from Transgenic Tobacco Plants Using CRISPR/Cas9 System. Plant gene. 26, 100288. 10.1016/j.plgene.2021.100288

[B84] Rosellini D. (2012). Selectable Markers and Reporter Genes: A Well Furnished Toolbox for Plant Science and Genetic Engineering. Crit. Rev. Plant Sci. 5, 401–453. 10.1080/07352689.2012.683373

[B85] Sah S. K. Kaur A. Kaur G. Cheema G. S. (2014). Genetic Transformation of Rice: Problems, Progress and Prospects. J. Rice Res. 3, 132. 10.4172/2375-4338.1000132

[B86] Sawada K. Hasegawa M. Tokuda L. Kameyama J. Kodama O. Kohchi T. (2004). Enhanced Resistance to Blast Fungus and Bacterial Blight in Transgenic Rice Constitutively Expressing OsSBP, a Rice Homologue of Mammalian Selenium-Binding Proteins. Biosci. Biotechnol. Biochem. 68, 873–880. 10.1271/bbb.68.873 15118317

[B87] Sengupta S. Chakraborti D. Mondal H. A. Das S. (2010). Selectable Antibiotic Resistance Marker Gene-free Transgenic Rice Harboring the Garlic Leaf Lectin Gene Exhibits Resistance to Sap-Sucking Planthoppers. Plant Cell Rep. 29 (3), 261–271. 10.1007/s00299-010-0819-7 20094886

[B88] Shan Q. Zhang Y. Chen K. Zhang K. Gao C. (2015). Creation of Fragrant Rice by Targeted Knockout of the OsBADH2 Gene Using TALEN Technology. Plant Biotech. J. 13 (6), 791–800. 10.1111/pbi.12312 25599829

[B89] Singh N. Choudhury D. R. Tiwari G. Singh A. K. Kumar S. Srinivasan K. (2016). Genetic Diversity Trend in Indian Rice Varieties: an Analysis Using SSR Markers. BMC Genet. 17, 127. 10.1186/s12863-016-0437-7 27597653 PMC5011800

[B90] Singh P. K. Verma R. L. Singh R. Singh R. K. Singh H. B. Arsode P. (2020). “Biotic Stress Management in Rice (Oryza Sativa L.) through Conventional and Molecular Approaches,” in New Frontiers in Stress Management for Durable Agriculture. Editors Rakshit A. Singh H. Singh A. Singh U. Fraceto L. (Singapore: Springer), 609–644. 10.1007/978-981-15-1322-0-3010.1007/978-981-15-1322-0_30

[B91] Sreekala C. Wu L. Gu K. Wang D. Tian D. Yin Z. (2005). Excision of a Selectable Marker in Transgenic Rice (Oryza Sativa L.) Using a Chemically Regulated Cre/loxP System. Plant Cell Rep. 24 (2), 86–94. 10.1007/s00299-004-0909-5 15662501

[B92] Sripriya R. Raghupathy V. Veluthambi K. (2008). Generation of Selectable Marker-free Sheath Blight Resistant Transgenic Rice Plants by Efficient Cotransformation of a Cointegrate Vector T-DNA and a Binary Vector T-DNA in One Agrobacterium Tumefaciens Strain. Plant Cell Rep. 27 (10), 1635–1644. 10.1007/s00299-008-0586-x 18663452

[B93] Stallworth S. Schumaker B. Fuller M. G. Tseng T. M. (2020). “Consequences and Mitigation Strategies of Biotic and Abiotic Stress in Rice (Oryza Sativa L.),” in Plant Stress Physiology. Editor Hossain A. Intechopen, London. 10.5772/intechopen.91402

[B94] Svitashev S. Schwartz C. Lenderts B. Young J. K. Cigan A. M. (2016). Genome Editing in Maize Directed by CRISPR–Cas9 Ribonucleoprotein Complexes. Nat. Commun. 7, 13274. 10.1038/ncomms13274 27848933 PMC5116081

[B95] Tan J. Wang Y. Chen S. Lin Z. Zhao Y. Xue Y. (2022). An Efficient Marker Gene Excision Strategy Based on CRISPR/Cas9-mediated Homology-Directed Repair in Rice. Int. J. Mol. Sci. 23 (3), 1588. 10.3390/ijms23031588 35163510 PMC8835944

[B96] Toda E. Koiso N. Takebayashi A. Ichikawa M. Kiba T. Osakabe K. (2019). An Efficient DNA-And Selectable-marker-free Genome-Editing System Using Zygotes in Rice. Nat. Plants 5 (4), 363–368. 10.1038/s41477-019-0386-z 30911123

[B97] Tu J. Datta K. Oliva N. Zhang G. Xu C. Khush G. S. (2003). Site‐independently Integrated Transgenes in the Elite Restorer Rice Line Minghui 63 Allow Removal of a Selectable Marker from the Gene of Interest by Self‐segregation. Plant Biotechnol. J. 1 (3), 155–165. 10.1046/j.1467-7652.2003.00012.x 17156029

[B98] Turnbull C. Lillemo M. Hvoslef-Eide T. A. K. (2021). Global Regulation of Genetically Modified Crops and the Gene Edited Crop Boom – A Review. Front. Plant Sci. 12, 630396. 10.3389/fpls.2021.630396 33719302 PMC7943453

[B99] Twyman R. M. Stoger E. Kohli A. Capell T. Christou P. (2002). “Selectable and Screenable Markers for Rice Transformation,” in Testing for Genetic Manipulation in Plants. Molecular Methods of Plant Analysis. Editors Jackson J. F. Linskens H. F. (Berlin, Heidelberg: Springer), 22. 10.1007/978-3-662-04904-4_1

[B100] Wang M. Lu Y. Botella J. R. Mao Y. Hua K. Zhu J.-,k. (2017). Gene Targeting by Homology-Directed Repair in Rice Using a Geminivirus-Based CRISPR/Cas9 System. Mol. Plant. 10, 1007–1010. 10.1016/j.molp.2017.03.002 28315751

[B101] Waseem M. Huang F. Wang Q. Aslam M. M. Abbas F. Ahmad F. (2021). Identification, Methylation Profiling, and Expression Analysis of Stress-Responsive Cytochrome P450 Genes in Rice under Abiotic and Phytohormones Stresses. GM Crops Food 12 (1), 551–563. 10.1080/2F21645698.2021.190881310.1080/21645698.2021.1908813 33877001 PMC8820252

[B102] Woo H. J. Qin Y. Park S. Y. Park S. K. Cho Y. G. Shin K. S. (2015). Development of Selectable Marker-free Transgenic Rice Plants with Enhanced Seed Tocopherol Content through FLP/FRT-mediated Spontaneous Auto-Excision. PLoS One 10 (7), e0132667. 10.1371/journal.pone.0132667 26172549 PMC4501831

[B103] Xia Z. H. Li X. B. Chen C. Y. Fan H. K. Jiang G. H. Zhu L. H. (2006). Generation of Selectable Marker-free and Vector Backbone Sequence-free Xa21 Transgenic Rice. Sheng Wu Gong Cheng Xue Bao 22, 204–210. 16607944

[B104] Xu H. Yu M. Yin Y. Zhu C. Ji W. Zhang C. (2020). Generation of Selectable Marker-free Soft Transgenic Rice with Transparent Kernels by Downregulation of SSSII-2. Crop J 8 (1), 53–61. 10.1016/j.cj.2019.05.006

[B105] Xu M. Zhao S. Zhang Y. Yin H. Peng X. Cheng Z. (2017). Production of Marker-free Transgenic Rice (Oryza Sativa L.) with Improved Nutritive Quality Expressing AmA1. Iran. J. Biotechnol. 15 (2), 102. 10.15171/2Fijb.152710.15171/ijb.1527 PMC5811051 29845057

[B106] Yang A. Su Q. An L. Liu J. Wu W. Qiu Z. (2009). Detection of Vector- and Selectable Marker-free Transgenic Maize with a Linear GFP Cassette Transformation via the Pollen-Tube Pathway. J. Biotechnol. 139, 1–5. 10.1016/j.jbiotec10.1016/j.jbiotec.2008.08.012 18831993

[B107] Yarasi B. Sadumpati V. Immanni C. P. Vudem D. R. Khareedu V. R. (2008). Transgenic Rice Expressing Allium Sativum Leaf Agglutinin (ASAL) Exhibits High-Level Resistance against Major Sap-Sucking Pests. BMC Plant Biol. 8, 102. 10.1186/1471-2229-8-102 18854007 PMC2579298

[B108] Yau Y. Y. Stewart C. N. (2013). Less Is More: Strategies to Remove Marker Genes from Transgenic Plants. BMC Biotechnol. 13, 36. 10.1186/1472-6750-13-36 23617583 PMC3689633

[B109] Yu H. X. Liu Q. Q. Ling W. A. N. G. Zhao Z. P. Li X. U. Huang B. L. (2006). Breeding of Selectable Marker-free Transgenic Rice Lines Containing AP1 Gene with Enhanced Disease Resistance. Agri. Sci. China 5 (11), 805–811. 10.1016/s1671-2927(06)60128-4

[B110] Yu H. X. Liu Q. Q. Xu L. Lu M. F. Yang X. J. Gong Z. Y. (2009). Quality Characteristics and Field Performance of Selectable Marker-free Transgenic Rice with Antisense Wx Gene and Improved Quality Derived from the Elite Parents of Hybrid Indica Rice. J. Cereal Sci. 50 (3), 370–375. 10.1016/j.jcs.2009.07.003

[B111] Yu H. Yao Q. Wang L. Zhao Z. Gong Z. Tang S. (2009). Generation of Selectable Marker-free Transgenic Rice Resistant to Chewing Insects Using Two Co-transformation Systems. Prog. Nat. Sci. 19 (11), 1485–1492. 10.1016/j.pnsc.2009.04.005

[B112] Zafar K. Sedeek K. E. M. Rao G. S. Khan M. Z. Amin I. Kamel R. (2020). Genome Editing Technologies for Rice Improvement: Progress, Prospects, and Safety Concerns. Front. Genome Ed. 2, 5. 10.3389/fgeed.2020.00005 34713214 PMC8525367

[B113] Zhang L. Zuris J. A. Viswanathan R. Edelstein J. N. Turk R. Thommandru B. (2021). AsCas12a Ultra Nuclease Facilitates the Rapid Generation of Therapeutic Cell Medicines. Nat. Commun. 2 (1), 3908. 10.1038/s41467-021-24017-8 PMC8222333 34162850

[B114] Zhao Y. Qian Q. Wang H. Z. Huang D. N. (2007). Co-transformation of Gene Expression Cassettes via Particle Bombardment to Generate Safe Transgenic Plant without Any Unwanted DNA. *In Vitro* Cell. Dev. Biol. 43 (4), 328–334. 10.1007/s11627-007-9051-8

[B115] Zhu Q. Yu S. Zeng D. Liu H. Wang H. Yang Z. (2017). Development of “Purple Endosperm Rice” by Engineering Anthocyanin Biosynthesis in the Endosperm with a High-Efficiency Transgene Stacking System. Mol. Plant 10 (7), 918–929. 10.1016/j.molp.2017.05.008 28666688

